# Bladder Morbidity and Hepatic Fibrosis in Mixed *Schistosoma haematobium* and *S. mansoni* Infections: A Population-Wide Study in Northern Senegal

**DOI:** 10.1371/journal.pntd.0001829

**Published:** 2012-09-27

**Authors:** Lynn Meurs, Moustapha Mbow, Kim Vereecken, Joris Menten, Souleymane Mboup, Katja Polman

**Affiliations:** 1 Institute of Tropical Medicine, Antwerp, Belgium; 2 Laboratory of Bacteriology and Virology, Centre Hospitalier Universitaire Aristide Le Dantec, Dakar, Senegal; Ministry of Health, Uganda

## Abstract

**Background:**

The global distribution map of schistosomiasis shows a large overlap of *Schistosoma haematobium*- and *S. mansoni*-endemic areas in Africa. Yet, little is known about the consequences of mixed *Schistosoma* infections for the human host. A recent study in two neighboring co-endemic communities in Senegal indicated that infection intensities of both species were higher in mixed than in single infections. Here, we investigated the relationship between mixed *Schistosoma* infections and morbidity in the same population. So far, this has only been studied in children.

**Methods:**

*Schistosoma* infection was assessed by microscopy. *Schistosoma*-specific morbidity was assessed by ultrasound according to WHO guidelines. Multivariable logistic regression models were used to identify independent risk factors for morbidity.

**Principal Findings:**

Complete parasitological and morbidity data were obtained from 403 individuals. *Schistosoma haematobium*-specific bladder morbidity was observed in 83% and *S. mansoni*-specific hepatic fibrosis in 27% of the participants. Bladder morbidity was positively associated with *S. haematobium* infection intensity (OR = 1.9 (95% CI 1.3–2.9) for a 10-fold increase in intensity). Moreover, people with mixed infections tended to have less bladder morbidity than those with single *S. haematobium* infections (OR = 0.3 (95% CI 0.1–1.1)). This effect appeared to be related to ectopic *S. mansoni* egg elimination in urine. Hepatic fibrosis on the other hand was not related to *S. mansoni* infection intensity (OR = 0.9 (95% CI 0.6–1.3)), nor to mixed infections (OR = 1.0 (95% CI 0.7–1.7)).

**Conclusions/Significance:**

This is the first population-wide study on the relationship between mixed *Schistosoma* infections and morbidity. Mixed infections did not increase the risk of *S. mansoni*-associated morbidity. They even tended to reduce the risk of *S. haematobium*-associated morbidity, suggesting a protective effect of *S. mansoni* infection on bladder morbidity. These unexpected results may have important consequences for schistosomiasis control in co-endemic areas and warrant further investigation.

## Author Summary

In the developing world, over 207 million people are infected with parasitic *Schistosoma* worms. *Schistosoma haematobium* and *S. mansoni* are the most abundant species in Africa and many people carry both. Yet, little is known about the consequences of such mixed infections. In general, *S. haematobium* affects the urinary tract of the host and *S. mansoni* the liver. Here, we investigated the effect of mixed *Schistosoma* infection on these health problems. We examined 403 people from northern Senegal for *Schistosoma* infections as well as for abnormalities of the urinary bladder and liver. Recently, we observed that people with mixed *Schistosoma* infections had generally higher infection intensities than those with single infections. The present study showed that abnormalities of the urinary bladder were more common in heavy than in light *S. haematobium* infections. Also, they were more common in single *S. haematobium* than in mixed infections. So far, only two studies have looked into the relationship between mixed *Schistosoma* infection and abnormalities of the bladder and liver, but only investigated children. Our findings suggest a possible protective effect of *S. mansoni* on bladder disease, in children as well as in adults. This may have important consequences for schistosomiasis control in co-endemic areas.

## Introduction

Worldwide more than 207 million people are infected with *Schistosoma*, 85% of whom live in Africa [1]. Due to the large overlap in *Schistosoma haematobium*- and *S. mansoni*-endemic areas [2]–[4] many people are at risk of co-infection. Yet, little is known about mixed *Schistosoma* infections and their impact on host morbidity.

Clinical manifestations of schistosomiasis are associated with the species-specific oviposition sites [2]. Both *S. haematobium* and *S. mansoni* mature in the portal vein of the human host and form male-female pairs. Subsequently, the *S. mansoni* male carries the female to the mesenteric plexus whereas the *S. haematobium* couple continues its way to the veins of the pelvis. In these respective sites, they lay eggs, which are eventually eliminated from the body via the urine (*S. haematobium*) or feces (*S. mansoni*). About half of the eggs, however, are carried away with the blood stream and/or trapped in the tissues. These retained eggs provoke inflammatory and granulomatous immune responses [2]. For *S. haematobium*, this can lead to inflammation, ulceration and pseudopolyposis of bladder and ureteral walls, and in children this is often accompanied by hematuria. Chronic lesions may evolve to fibrotic and sandy patches with severe sequelae such as hydroureter, hydronephrosis, and squamous bladder cancer [2], [5], [6]. For *S. mansoni*, egg deposition can lead to inflammatory hepatic schistosomiasis and hepatosplenomegaly in children and adolescents [7]. Trapped schistosomal eggs are gradually replaced by fibrotic deposits, and give rise to chronic hepatic schistosomiasis [2].

In mixed *S. mansoni* and *S. haematobium* infections, the above-described processes act in parallel, which may result in more severe or other abnormalities than in single infections. So far, only two studies have investigated the relationship between mixed infections and host morbidity. In Zimbabwe, a positive association between *S. mansoni* egg output and liver size was found in the presence but not in the absence of *S. haematobium*. Yet, this effect was very little [8]. In a study in Mali, mixed infection was associated with reduced hepatic morbidity on one hand and increased bladder morbidity on the other [9]. Both studies were performed in schoolchildren, in whom severe hepatic schistosomiasis is unlikely to have developed already [2], [7], [10]. Community-wide studies would therefore be more appropriate to investigate mixed infections and morbidity.

In northern Senegal, many communities have in the past decades become co-endemic for *S. mansoni* and *S. haematobium* [11]–[16]. *Schistosoma mansoni* was introduced in Richard-Toll in 1988 upon construction of the Diama dam and rapidly spread throughout the region [17], [18]. By 1994, virtually the entire Guiers Lake (‘Lac de Guiers’) area had become exposed to this species [19]. Today, both *S. mansoni* and *S. haematobium* are widely spread, resulting in a large number of people with mixed infections in the communities around the lake.

Recently, we reported on the distribution of and risk factors for mixed *Schistosoma* infections in two communities on the banks of Lake Guiers. Individuals with mixed infections were found to have higher infection intensities than those with single infections [20]. In the present study, we set out to investigate the relationship between mixed *Schistosoma* infections and morbidity in the same communities. We studied the patterns of *S. haematobium*-specific bladder morbidity and *S. mansoni*-specific hepatic morbidity in this co-endemic focus, and compared morbidity in people with mixed *Schistosoma* infections to those with single infections.

## Methods

### Ethics Statement

This study was part of a larger investigation on the epidemiology of schistosomiasis and innate immune responses (SCHISTOINIR: www.york.ac.uk/res/schistoinir) for which approval was obtained from the review board of the Institute of Tropical Medicine, the ethical committee of the Antwerp University Hospital and ‘Le Comité National d'Ethique de la Recherche en Santé’ in Dakar. Informed and written consent was obtained from all participants prior to inclusion into the study.

### Study Area

This study was conducted from July until November 2009 in Ndieumeul (also known as Thiekène) and Diokhor Tack, two neighboring communities on the Nouk Pomo peninsula in Lake Guiers. Details on the study area have been described elsewhere [20].

### Parasitology and Urine Dipstick

Two urine and two feces samples were collected from each participant on consecutive days for microscopic analysis [21], [22]. Per feces sample, two Kato-Katz slides of 25 mg fecal material each were prepared and microscopically examined for *Schistosoma* species [22]. *Schistosoma mansoni* infection intensity was expressed as the number of eggs detected per gram of feces (epg). Urine filtration was performed using a filter of 12 µm pore-size (Isopore) according to standard procedures [21]. *Schistosoma haematobium* infection intensity was expressed as the number of eggs detected per 10 ml of urine (ep10ml). Ectopic eggs were measured qualitatively (positive/negative). Ectopic egg elimination refers to elimination of schistosomal eggs via the unusual route – i.e. *S. mansoni* eggs in urine or *S. haematobium* eggs in feces. Single infection was defined as passing eggs of only one species, and mixed infection as passing eggs of both *S. mansoni* and *S. haematobium*, regardless of the route of egg elimination [20]. Microhematuria was determined in a subsample using Combur 7 dipsticks (Roche) on the first urine sample. All community members were offered praziquantel (one dose of 40 mg/kg body weight) and mebendazole (one dose of 500 mg) treatment after the study according to WHO guidelines [23].

### Ultrasound

Participants were examined using a portable ultrasonography device with convex transducer. Pathologic lesions associated with *S. haematobium* or *S. mansoni* infection were recorded according to the Niamey guidelines [10]. All examinations were performed by the same clinician who was blind to the participant's infection status. Participants with severe pathology that needed further treatment were referred to the appropriate health authority. For *S. haematobium*-specific morbidity, the urinary bladder score was determined [10]. A score of ≥1 was considered as *S. haematobium*-specific urinary bladder morbidity in accordance with previous studies [9], [24]–[30]. Individuals with a score of 0 were categorized as controls. For *S. mansoni*-specific morbidity, the liver image pattern was determined [10]. Additional measurement of periportal thickening was not included as this approach has been shown to be not reproducible [31]. Liver image patterns of C to F were categorized as *S. mansoni*-specific hepatic morbidity [31]–[35]. Individuals with liver image pattern A or B are not likely to have periportal fibrosis [10] and were therefore categorized as controls. Individuals with signs of hepatic morbidity that were not specific for *S. mansoni* (e.g. hepatitis, cirrhosis or fatty liver) were excluded [10].

### Statistical Analysis

IBM SPSS 19.0 (SPSS, Inc.) was used for statistical analysis. Results were considered significant when the *p*-value was <0.05. As egg outputs showed skewed distributions, data were normalized by log (base 10)-transformation after adding half of the detection limit to allow for zeros.

Differences between groups were determined by the Pearson Chi-square test for community and gender, and by the Mann-Whitney U test for age. Furthermore, the Pearson Chi-square test was used to determine the association between bladder morbidity and microhematuria, as well as between bladder and liver morbidity.

Because of the non-linear trend of morbidity over age, the population was divided into four age groups (0–9, 10–19, 20–39 and ≥40 years) for multivariable regression analysis. Multivariable logistic regression models were used to identify independent risk factors for *S. haematobium*-specific bladder morbidity and *S. mansoni*-specific hepatic fibrosis, respectively. Age, gender, community of residence, *S. haematobium* infection intensity and *S. mansoni* infection intensity were included as potential risk factors. Moreover, significant interaction terms with age (*p*<0.05) were added.

Similar models were used to assess the independent effect of mixed infection (as compared to single infection) on *S. haematobium*-specific bladder morbidity and *S. mansoni*-specific hepatic fibrosis, respectively. Among *S. haematobium*-positive subjects, the association between bladder morbidity and mixed infection was investigated using a dummy variable for mixed infection (1 = mixed, 0 = single), and age, gender, community of residence and *S. haematobium* infection intensity as other determinants. Likewise, the association between hepatic fibrosis and mixed infection was investigated in *S. mansoni*-positive subjects with a dummy variable for mixed infection and upon correction for age, gender, community of residence and *S. mansoni* infection intensity.

## Results

Complete parasitological data were obtained from 857 individuals [20]. Ultrasound data were collected from a random subsample of 403 individuals. The latter group consisted of 207 males and 196 females with a median age of 16 (range 3–85) years. There were no significant dissimilarities between those who participated in the ultrasound examination and those who did not, except for a slightly lower percentage of individuals participating from the community of Ndieumeul as compared to Diokhor Tack (*p* = 0.005), and from the youngest as compared to the older age groups (*p* = 0.030). No significant differences in age or gender were observed between the two communities.

### Morbidity Prevalences

*Schistosoma haematobium*-specific bladder morbidity was observed in 83% of the study population (334/403; Table 1). Most common lesions concerned multifocal or diffuse bladder wall thickening (n = 189), irregularities (n = 94) or a single mass (n = 19). Microhematuria was twice as prevalent among those with bladder morbidity as compared to those without (44% *versus* 21%, *p* = 0.002).

**Table 1:** *Schistosoma haematobium*-associated bladder morbidity, hematuria and *S. haematobium* infection in the two co-endemic communities studied.

Morbidity	Urinary bladder score	n	(%)	Microhematuria (%)	*S. haematobium* infection	
					%	GM (95%CI)^a^
**Negative**	**0**	**69**	**(17)**	**21**	**33**	**3.9 (1.8–8.1) ep10ml**
**Positive**	**≥1**	**334**	**(83)**	**44**	**58**	**8.8 (6.7–11.5) ep10ml**
	1	15	(4)	62	73	8.5 (2.2–31.5) ep10ml
	2	301	(75)	42	56	8.5 (6.4–11.3) ep10ml
	≥3	18	(4)	71	78	13.1 (4.2–39.5) ep10ml
**Total**		**403**	**(100)**	**40**	**54**	**8.1 (6.2–10.4) ep10ml**

a GM) Geometric Mean; calculated for microscopically *S. haematobium*-positive individuals only.

*S. mansoni*-specific fibrosis was present in 27% (109/403) of the population (Table 2). Liver image patterns up to F were observed, but the large majority had pattern C (89/109).

**Table 2:** *Schistosoma mansoni*-associated hepatic fibrosis and *S. mansoni* infection in the two co-endemic communities studied.

Morbidity	Liver image pattern	n	(%)	*S. mansoni* infection	
				%	GM (95%CI)^a^
**Negative**	**A–B**	**282**	**(70)**	**72**	**123 (99–153) epg**
	A	142	(35)	75	131 (98–175) epg
	B	140	(35)	69	115 (83–160) epg
**Positive**	**C–F**	**109**	**(27)**	**61**	**113 (76–168) epg**
	C	89	(22)	63	125 (81–193) epg
	D	10	(2)	40	113 (29–400) epg
	E	9	(2)	67	45 (10–160) epg
	F	1	(0.2)	0	N/A
**Excluded**		**12**	**(3)**		
**Total**		**403**	**(100)**	**69**	**120 (99–145) epg**

a GM) Geometric Mean; calculated for microscopically *S. mansoni*-positive individuals only. N/A) Not Applicable.

Morbidity of both liver and bladder was observed in 24% (93/391) of the study participants (Table 3). Those who had bladder morbidity tended to be more at risk for hepatic fibrosis and *vice versa* (odds ratio (OR) = 1.3 (95% confidence interval (CI) 0.7–2.4)).

**Table 3:** Bladder and liver co-morbidity in the two co-endemic communities studied.

Bladder morbidity	Hepatic fibrosis		
	Negative	Positive	Total
**Negative**	51	16	67
**Positive**	231	93	324
**Total**	282	109	391

Number of cases.

### Age-Related Patterns

**Figure 1:**
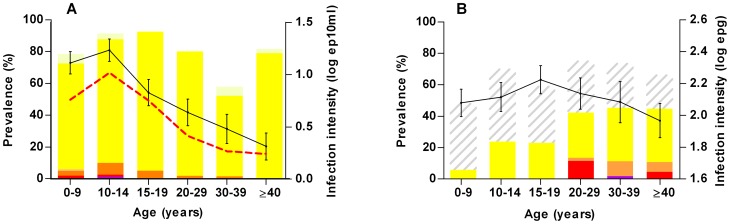
Age distribution of schistosomiasis morbidity in the two co-endemic communities studied. Colored stacks indicate morbidity prevalences and continuous black lines indicate mean 10log-transformed infection intensities among positive subjects with the standard error of the mean (whiskers). **Panel A:** Different forms of *S. haematobium*-specific bladder morbidity are denoted by a color gradient: light yellow stacks designate a urinary bladder score of 1, bright yellow a score of 2 and orange (3 and 4), red (5) and violet (6) indicate higher morbidity scores. The dotted red line indicates hematuria prevalence in a subsample (n = 317). **Panel B:** The severity of *S. mansoni*-specific fibrosis is denoted by a color gradient. Yellow stacks designate liver image pattern C, orange pattern D, red pattern E, and violet stacks indicate pattern F. Striped stacks designate those with borderline liver morbidity (pattern B, not classified as morbidity).

Figure 1 shows that bladder morbidity was mainly observed in children (<20 years), with a peak in 10-to-19-year-olds. The age-related distribution of bladder morbidity coincided with that of *S. haematobium* infection intensity and microhematuria, although the peak was slightly later in adolescence, and the subsequent decline in adults less pronounced.

The prevalence of hepatic fibrosis increased from 6% in 0-to-9-year-olds to 44% in those ≥20 years old. The more severe forms of hepatic fibrosis (liver image patterns D, E and F) only became apparent in adults (≥20 years old). The peak in the age-related distribution of hepatic fibrosis occurred more than 10 years later in life than the *S. mansoni* infection intensity peak (Figure 1).

### Risk Factors

Multivariable analysis showed age to be a significant risk factor for bladder morbidity as well as hepatic fibrosis (Table 4). However, age-related patterns of hepatic fibrosis differed between the two communities (*p* = 0.033): while the ORs for hepatic fibrosis increased with age in Diokhor Tack (*p*<0.001), they did not vary with age in Ndieumeul (data not shown). Individuals from Diokhor Tack were significantly more at risk for hepatic fibrosis but tended to be less at risk for bladder morbidity than their counterparts from Ndieumeul (Table 4). Furthermore, females were less at risk for both forms of morbidity than males. Neither *S. mansoni* intensity nor *S. haematobium* intensity was identified as an independent risk factor for hepatic fibrosis. On the other hand, *S. haematobium* (but not *S. mansoni*) infection intensity was a strong risk factor for bladder morbidity.

**Table 4:** Risk factors for schistosomiasis morbidity in the total study population.

Risk factors		*S. haematobium*-specific bladder morbidity (n = 403)			*S. mansoni*-specific hepatic fibrosis (n = 391)		
		n	Unadjusted OR (95% CI)	Adjusted OR (95% CI)	n	Unadjusted OR (95%CI)	Adjusted OR (95%CI)
Age^a^	<10 y	102	0.3 (0.1–0.7)**	0.3 (0.1–0.8)*	102	0.1 (0.03–0.2)***	0.1 (0.01–1.3)
	10–19 y	121	Ref.	Ref.	119	0.4 (0.2–0.7)**	1.4 (0.4–5.1)
	20–39 y	109	0.3 (0.1–0.7)**	0.6 (0.3–1.3)	105	Ref.	Ref.
	≥40 y	71	0.4 (0.2–0.97)*	0.9 (0.3–2.3)	65	1.0 (0.6–1.9)	0.7 (0.1–3.8)
Gender	Male	207	Ref.	Ref.	200	Ref.	Ref.
	Female	196	0.4 (0.2–0.7)***	0.3 (0.2–0.6)***	191	0.8 (0.5–1.3)	0.5 (0.3–0.9)*
Community	Ndieumeul	100	Ref.	Ref.	99	Ref.	Ref.
	Diokhor Tack	303	0.4 (0.2–0.8)*	0.6 (0.3–1.3)	292	1.9 (1.1–3.3)*	5.1 (1.3–20.8)*
*S. haematobium*	Infection intensity^b^	403	2.0 (1.4–2.9)***	1.9 (1.3–2.9)**	391	0.6 (0.5–0.8)***	0.9 (0.7–1.3)
*S. mansoni*	Infection intensity^b^	403	1.5 (1.1–2.1)*	1.1 (0.8–1.6)	391	0.8 (0.6–1.0)	0.9 (0.6–1.3)
Interaction	Age×community	N/A	N/A	N/A	391	N/A	*p* = 0.033^a^

OR) Odds Ratio; 95%CI) 95% Confidence Interval; Ref.) Reference category; N/A) Not Applicable; *) *p*<0.05; **) *p*<0.01; ***) *p*<0.001.

a For *S. haematobium*-specific bladder morbidity, the trend with age was significant at the level of *p* = 0.025 in the uni- and *p* = 0.043 in the multivariable analysis. For *S. mansoni*-specific hepatic fibrosis, the trend with age was significant in the crude analysis (*p*<0.001). In the adjusted analysis the ORs for hepatic fibrosis increased with age in Diokhor Tack (*p*<0.001) but they did not vary with age in Ndieumeul.

b OR for a 10-fold increase in infection intensity.

### Effect of Mixed Infection

After including mixed infection into the multivariable model, the above-described trends remained the same (Table 5). The risk of hepatic fibrosis did not differ between subjects with single *S. mansoni* and those with mixed infections. Interestingly however, mixed infection tended to be negatively associated with *S. haematobium*-specific bladder morbidity, suggesting a protective effect of current *S. mansoni* infection (*p* = 0.068).

**Table 5:** The effect of mixed *Schistosoma* infection on bladder morbidity and on hepatic fibrosis.

Risk factors		Bladder morbidity in *S. haematobium*-infected subjects (n = 216)			Hepatic fibrosis in *S. mansoni*-infected subjects (n = 270)		
		n	Unadjusted OR (95% CI)	Adjusted OR (95% CI)	n	Unadjusted OR (95%CI)	Adjusted OR (95%CI)
Age^a^	<10 y	63	0.3 (0.1–1.1)	0.2 (0.1–0.9)*	65	0.1 (0.03–0.3)***	0.1 (0.02–0.2)***
	10–19 y	88	Ref.	Ref.	102	0.4 (0.2–0.8)**	0.3 (0.2–0.7)**
	20–39 y	44	0.2 (0.1–0.8)*	0.3 (0.1–1.2)	66	Ref.	Ref.
	≥40 y	21	0.3 (0.1–1.4)	0.5 (0.1–3.0)	37	0.8 (0.3–1.8)	0.8 (0.4–1.9)
Gender	Male	109	Ref.	Ref.	140	Ref.	Ref.
	Female	107	0.3 (0.1–0.8)*	0.3 (0.1–0.9)*	130	1.0 (0.6–1.8)	0.6 (0.3–1.2)
Community	Ndieumeul	70	Ref.	Ref.	87	Ref.	Ref.
	Diokhor Tack	146	0.2 (0.04–0.8)*	0.2 (0.04–0.7)*	183	1.5 (0.8–2.8)	1.6 (0.8–3.2)
*S. haematobium*	Infection intensity^b^	216	1.8 (0.96–3.3)	1.8 (0.9–3.7)		N/A	N/A
*S. mansoni*	Infection intensity^b^		N/A	N/A	270	0.9 (0.6–1.4)	1.0 (0.7–1.7)
Mixed infection	No	40	Ref.^c^	Ref.^c^	97	Ref.^d^	Ref.^d^
	Yes	176	0.6 (0.2–2.2)	0.3 (0.1–1.1)	173	0.8 (0.6–1.1)	1.1 (0.8–1.5)

OR) Odds Ratio; 95%CI) 95% Confidence Interval; Ref.) Reference category; N/A) Not Applicable; *) *p*<0.05; **) *p*<0.01; ***) *p*<0.001.

a The trends with age were not significant for *S. haematobium*-specific bladder morbidity, but for *S. mansoni*-specific hepatic fibrosis, they were at the level of *p*<0.001 in both analyses.

b OR for a 10-fold increase in infection intensity.

c Mixed infections as compared to single *S. haematobium* infections.

d Mixed infections as compared to single *S. mansoni* infections.

### Ectopic Egg Elimination and Bladder Morbidity

Ectopic *S. haematobium* eggs were found in one (0.6%) and ectopic *S. mansoni* eggs in 23 (13%) out of 176 individuals with mixed infections. Table 6 illustrates the importance of the route of *S. mansoni* egg elimination in the development of bladder morbidity. Those who eliminated *S. mansoni* via both urine and feces (n = 17) had highest *S. haematobium* infection intensities and prevalences of bladder morbidity. Lowest prevalences of morbidity were observed in those who exclusively eliminated *S. mansoni* eggs via the urine (and not via the feces; n = 6), despite relatively high *S. haematobium* infection intensities.

**Table 6:** *S. mansoni* egg elimination, infection intensity and bladder morbidity in subjects passing *S. haematobium* eggs in urine.

Route of *S. mansoni* egg elimination		n	*S. haematobium* infection intensity (GM (95% CI))^a^	Bladder morbidity (%)
No elimination	(single *S. haematobium* infection)	39	3.1 (2.0–4.7) ep10ml	92
Exclusively via feces	(mixed infection)	153	8.0 (6.0–10.8) ep10ml	89
Via feces & urine	(mixed infection)	17	60.1 (27.2–132.3) ep10ml	100
Exclusively via urine	(mixed infection)	6	12.0 (1.5–86.5) ep10ml	50
**Total**		**215**	**8.1 (6.2–10.4) ep10ml**	89

a GM) Geometric Mean; 95%CI) 95% Confidence Interval.

## Discussion

The global distribution map of schistosomiasis shows a large overlap of *Schistosoma mansoni*- and *S. haematobium*-endemic areas in Africa [2]–[4]. Yet, little is known about the consequences of mixed *Schistosoma* infections for the human host. Here, we report on the relationship of mixed *Schistosoma* infections with *S. haematobium*-specific bladder morbidity and *S. mansoni*-specific hepatic morbidity in two co-endemic communities.

Risk factors for schistosomal morbidity in this co-endemic area – i.e. age, gender, community of residence – were similar to those generally observed in *Schistosoma*-endemic areas. In line with other studies in mono-endemic areas, we observed that children are more at risk of bladder morbidity [36], [37], while adults are more at risk for hepatic fibrosis [2], [38]. Participants from one community tended to be less at risk for bladder morbidity but were significantly more at risk for hepatic fibrosis than participants from the other. Micro-geographical differences (i.e. between neighboring communities) have been reported before [32]–[34], [39], and are probably due to geographical heterogeneities in *Schistosoma* exposure (history) [20], [40]–[42], although other factors (e.g. genetic or environmental factors) may also be involved [39]. Males were more at risk for bladder morbidity and hepatic fibrosis than females. Previous studies in either *S. mansoni*- or *S. haematobium*-endemic areas have found similar associations for both forms of morbidity [25], [30]–[34], [36], [38], [43]–[49]. Possibly, males are more prone to *Schistosoma*-associated pathology because of hormonal and immunological differences. It has been proposed that hepatic fibrosis might be more pronounced in men because androgens can reduce interferon (IFN)-γ levels while estrogens have the opposite effect on this antifibrogenic cytokine [43]. Similarly, a study in Uganda showed that the risk of hepatic fibrosis was associated with different cytokine profiles in men and women [35].

Recently, we found infection intensities of both *S. mansoni* and *S. haematobium* to be elevated in mixed as compared to single infections [20]. In the present study, *S. haematobium* infection intensity was identified as an independent risk factor for bladder morbidity, in accordance with previous studies from mono-endemic areas [29], [30], [36], [44]–[48], [50]. However, subjects with mixed infections tended to have less bladder morbidity than those with single *S. haematobium* infections, independently of infection intensity. This would suggest a protective effect of *S. mansoni* on bladder morbidity.

The observed reduction in bladder morbidity in mixed infections appeared to be related to ectopic *S. mansoni* egg elimination. Lowest prevalences of bladder morbidity were observed in *S. haematobium*-positive individuals who also eliminated *S. mansoni* eggs in urine (but not in feces), despite the relatively high *S. haematobium* infection intensities in this group. Experimental models have shown that *S. mansoni* and *S. haematobium* can form heterologous male-female pairs. As the *S. haematobium* male is assumed to be competitively stronger than *S. mansoni* [51], [52], this would result in more heterologous pairs in the vesical than in the mesenteric plexus and thus in more ectopic *S. mansoni* than ectopic *S. haematobium* egg elimination in mixed foci [12], [20], [51]–[55]. Nothing is known yet about the pathogenicity of eggs from heterologous pairs [54], [56]. It could be speculated however, that these heterologous pairs would produce less (pathogenic) eggs than homologous *S. haematobium* worm pairs, or that their eggs would deviate to other sites [57], [58]. Competition between *S. mansoni* and *S. haematobium* females for *S. haematobium* males would then lead to a reduction of bladder morbidity in mixed as opposed to single infections. Other factors may also underlie the potential protective effect of *S. mansoni* infection. As our study population has been exposed to *S. mansoni* for a longer period of time than to *S. haematobium* [19], one could argue that *S. mansoni*-induced cross-resistance to *S. haematobium* might have played a role as well [59]. Obviously, more research is needed to confirm the observed relation between mixed *Schistosoma* infection and bladder morbidity, and to understand the underlying mechanisms.

Only one epidemiological study has looked into mixed infections and bladder morbidity before. In contrast to our study, the authors reported a positive association between mixed infection and bladder morbidity in Malian subjects [9]. However, they studied only schoolchildren (7–14 years) and did not take ectopic egg elimination into account. Restricting our analysis to children between 5 and 14 years (the limited sample size did not allow us to perform the analysis on a smaller age range), the association between mixed infection and bladder morbidity still tended to be negative (OR = 0.5 (95%CI 0.1–3.4)). Disregarding ectopic eggs as well, the association became positive (OR = 1.7 (95%CI 0.4–7.6)), which is in line with the Malian study (data not shown). These discrepancies clearly demonstrate the importance of considering ectopic egg elimination in mixed *Schistosoma* infections and morbidity.

In contrast to our findings for bladder morbidity, hepatic fibrosis was neither associated with current *S. mansoni* infection intensity nor with mixed infections. Hepatic fibrosis only develops after 5–15 years of exposure to *S. mansoni* [2]. This was illustrated by the >10 years' time lag between the *S. mansoni* infection and *S. mansoni*-associated hepatic fibrosis peaks in the respective age-related curves (Figure 1). While inflammatory hepatic morbidity is generally positively associated with current *S. mansoni* infection [2], [60], [61], the progression of morbidity into chronic schistosomiasis is driven by cumulative exposure to *S. mansoni* eggs rather than current infection [7], [62], [63].

So far, only two other studies have investigated the relationship between mixed infections and liver morbidity, with contrasting results. A Zimbabwean study found a slight positive association between mixed infections and liver size [8]. The afore-mentioned Malian study observed a negative association between mixed infections and hepatic fibrosis [9]. However, the latter classified liver image pattern B as abnormal [24], which is not according to the Niamey guidelines [10], [31]–[35], and hampers an adequate comparison with our findings. Adopting the criteria of the Malian study, we still found a positive association between mixed infections and liver fibrosis in children (7–14 years), although not significant (data not shown). The divergences between the Malian study and our study – for bladder as well as liver morbidity – could furthermore be due to differences in the distribution of *S. haematobium* and *S. mansoni* infection status and intensity between the three Malian study areas, which were not accounted for [9]. Also other differences in e.g. transmission dynamics [39], between the Malian and present study cannot be excluded.

### Conclusion

Up to now, the relationship between mixed *Schistosoma* infection and morbidity has only been studied in schoolchildren. This population-wide study is the first to include adults. Mixed infections were not associated with an increased risk of *S. mansoni*-associated morbidity and even tended to reduce the risk of bladder morbidity. These unexpected results warrant further investigation of a possible protective effect of *S. mansoni* on bladder morbidity. Especially the role of interspecies interactions and ectopic *S. mansoni* egg elimination should be studied in more detail, as these phenomena may have important consequences for schistosomiasis morbidity and control in co-endemic areas.
